# Slurry Erosion–Corrosion Characteristics of As-Built Ti-6Al-4V Manufactured by Selective Laser Melting

**DOI:** 10.3390/ma13183967

**Published:** 2020-09-08

**Authors:** Saleh Ahmed Aldahash, Osama Abdelaal, Yasser Abdelrhman

**Affiliations:** 1 Department of Mechanical and Industrial Engineering, College of Engineering, Majmaah University, Al-Majmaah 11952, Saudi Arabia; saldahash@mu.edu.sa; 2 Mechanical Engineering Department, Faculty of Engineering, Assiut University, Assiut 71516, Egypt; yasser.abdelrhman@aun.edu.eg

**Keywords:** selective laser melting (SLM), Ti-6Al-4V, slurry erosion, slurry erosion–corrosion, wear resistance, erosion mechanisms

## Abstract

Erosion and erosion–corrosion tests of as-built Ti-6Al-4V manufactured by Selective Laser Melting were investigated using slurries composed of SiO_2_ sand particles and either tap water (pure water) or 3.5% NaCl solution (artificial seawater). The microhardness value of selective laser melting (SLM)ed Ti-6Al-4V alloy increased as the impact angle increased. The synergistic effect of corrosion and erosion in seawater is always higher than erosion in pure water at all impact angles. The seawater environment caused the dissolution of vanadium oxide V_2_O_5_ on the surface of SLMed Ti-6Al-4V alloy due to the presence of Cl^−^ ions in the seawater. These findings show lower microhardness values and high mass losses under the erosion–corrosion test compared to those under the erosion test at all impact angles.

## 1. Introduction

Ti-6Al-4V is the most broadly utilized titanium alloy as it currently covers about 50% of the worldwide production of titanium alloys [1,2,3]. It is a duplex ((hcp, α-Ti) + (bcc, β-Ti))-type microstructure alloy at room temperature. The major advantages of Ti-6Al-4V are its high strength and hardness, low specific weight (roughly 45% lighter than steel), superior corrosion resistance, biocompatibility, high creep resistance, low maintenance cost, and long service life [4,5,6]. Thanks to these advantageous characteristics, Ti-6Al-4V has emerged as a powerful alloy for a wide field of applications. In addition to its wide use in surgical instruments and medical implants, it is currently used as a material for aerospace components (i.e., engine compressor blades and disks and helicopter rotor blades) [7,8,9], marine and offshore components (i.e., marine ship hulls, propellers, tubes, and shells) [1,10,11], oil and wastewater systems components (i.e., valves, pipelines, pipe fittings, and pumps) [12,13,14], hydropower plant components (i.e., turbine blades, pumps) [15,16,17], and architectural cladding and roofing [11]. These components are typically exposed to slurry erosion, where the working surfaces are continuously impacted by a stream of a slurry mixture. So, central to the entire discipline of Ti-6Al-4V industrial slurry carrier applications is the investigation of its tribological behaviors, especially slurry erosion and corrosion.

Slurry erosion is a type of mechanically induced wear process that occurs as a result of relative motion and/or impact between a stream of liquid containing suspended abrasive particles such as sand and the target surface, causing material degradation. It is a life-limiting challenge in the design of Ti-6Al-4V components exposed to a slurry environment leading to a drop in efficiency and increased maintenance cost. The characteristics of slurry erosion conditions include the material properties, liquid medium and erodent properties, slurry impact velocity, impact angle, and erodent concentration.

Casting, forging, machining, joining, and powder metallurgy are the dominant conventional processing techniques of Ti-6Al-4V [18,19]. Slurry erosion on conventionally processed Ti-6Al-4V has been studied by several authors under different conditions over the years [13,15,20,21,22,23,24,25,26]. The general outcome of all studies has been poor erosive wear resistance of Ti-6Al-4V, and it is recommended that hardness and yield strength should be enhanced to increase the erosion resistance. Surface coatings and heat treatments have been observed to be common practices to improve both surface hardening and strength. It was also reported that the Ti6Al4V alloy has ductile material erosion behavior with maximum erosive wear rate at an impact angle of around 20–30° [12,27,28,29].

The past few decades have seen increasingly rapid advances in the field of metal additive manufacturing (AM), making it a new manufacturing route for freeform fabrication of complex net-shape metal components with applications across a range of industrial sectors. In contrast to subtractive and formative manufacturing methods, such as turning and casting, AM allows the building of 3D objects layer-by-layer. The classification of AM techniques has been outlined elsewhere [30,31]. Selective laser melting (SLM) is one of the metal powder bed fusion AM processes. In SLM, objects are manufactured directly from specific metal powders in a layer-by-layer manner using a computer-controlled laser system following a digital design or acquisition and virtual slicing of complex 3D models. Ti-6Al-4V is the most common material used for SLM and it is currently being explored as a candidate material for many components where major failure occurs due to slurry erosive wear.

A layered manner and an extremely fast solidification rate (~10^3^ to 10^8^ K/s [32,33,34]) vs. (~20 to 100 K/s in casting [35]) during processing are important aspects of SLM that make it very different from conventional cast or wrought counterparts in terms of the microstructure and mechanical properties of products [36,37]. Previous research on the microstructure of as-built SLM-produced Ti-6Al-4V has indicated that brittle hexagonally packed acicular α’ martensitic structure contained within prior-β grains is formed due to the high cooling rate and smaller melt pools size during the melting process, resulting in higher strength and lower ductility, in contrast to the formation of coarse equiaxed β grains and straight (α + β) lamellas in typical as-cast Ti-6Al-4V [38,39,40,41,42,43]. Previous research also showed that the hardness of as-built SLMed Ti6Al4V is about (~ 430–450 HV) [44], which is higher than the hardness (~200 HV) of as-cast Ti-6Al-4V [45], as well as the hardness (~320–350 HV) of the superplastic forming process of Ti-6Al-4V [33]. In general, as-built Ti-6Al-4V processed via optimized SLM also has a comparable yield strength and ultimate tensile strength to conventionally processed counterparts; however, it has a higher surface roughness, lower ductility, and questionable fatigue performance [42,43,46,47,48,49,50].

In light of these differences, one of the major current concerns in SLMed Ti-6Al-4V is the investigation of its tribological behavior. Up to now, few studies have investigated the erosion behavior of additively manufactured Ti-6Al-4V [28]. Moreover, too little attention has been paid to the investigation of the slurry erosion/erosion–corrosion behavior of SLMed Ti-6Al-4V. The aim of the present study, therefore, is to experimentally investigate the slurry erosion and erosion–corrosion characteristics of as-built Ti-6Al-4V manufactured by selective laser melting using a slurry whirling arm rig under different conditions. The findings should make an important contribution to the field of tribology of SLMed Ti-6Al-4V parts.

## 2. Experimental Details

### 2.1. Material and Sample Preparation

**Figure 1:**
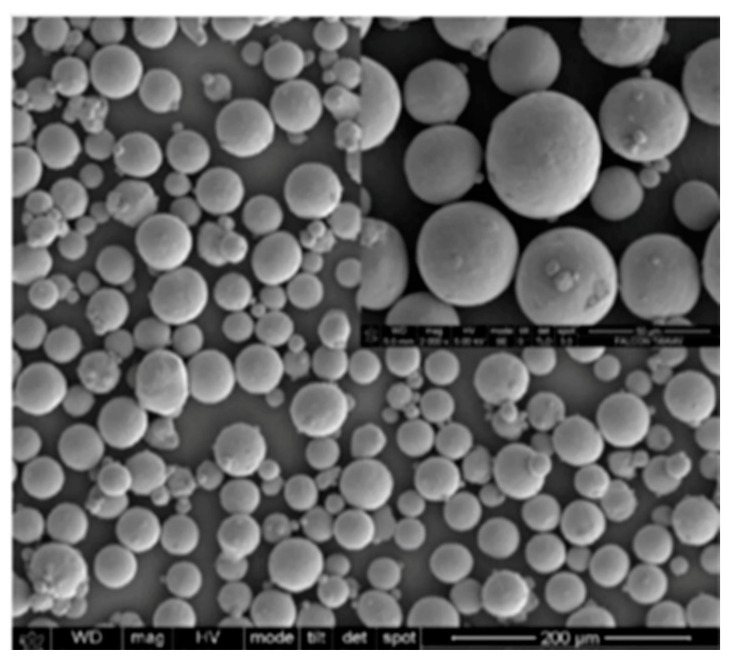
SEM micrograph of the surface morphology of powder used in the production of SLM specimens.

**Figure 2:**
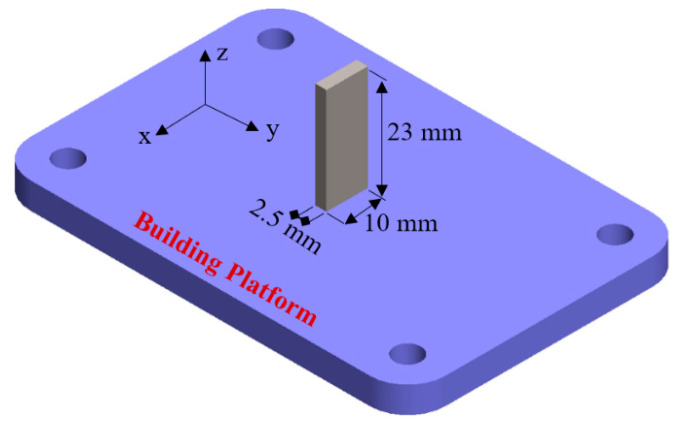
Schematic of the test sample. The x–z surfaces were exposed to the slurry stream.

The slurry erosion tests were performed on Ti-6Al-4V test samples shaped like rectangular blocks with dimensions of 23 mm × 10 mm × 2.5 mm. They were manufactured using the AM250 SLM system (Renishaw, Staffordshire, UK) from extra-low interstitial Ti-6Al-4V powder with particle sizes in the range of 15–100 µm and were sourced from Renishaw. The chemical composition of the powder before the manufacturing process is listed in Table 1. Figure 1 depicts an SEM image of the morphology of Ti-6Al-4V powder particles. Overall, particles have a smooth, spherical shape indicating a good flowability. The SLM processing parameters are shown in Table 2. Figure 2 shows the sample size and orientation on the building platform. After fabrication, the as-built SLMed samples were cut, and sample surfaces were subjected to standard metallographic procedures using the following protocol: grinding with 240, 600, 1200, and 2500 Grit SiC papers and then mirror polishing using a polishing cloth with suspensions containing 6, 3, and 1 μm diamond particles, followed by cleaning in distilled water. Next, to reveal the microstructure, selected polished samples were etched using a solution comprising 10% hydrofluoric acid (HF), 5% nitric acid (HNO_3_), and 85% distilled water for an etching time of 5–10 s. All samples were then degreased in ethanol using an ultrasound cleaner for 10 min each and dried using compressed hot air.

### 2.2. Microstructure and Microhardness Investigation

The surface morphology and microstructure of samples before and after erosion and erosion–corrosion tests were observed using both scanning electron microscopy (SEM, JEOL JSM 5400, Japan) and optical microscopy (PME OLYMPUS, Tokyo, Japan). Microhardness was measured on the eroded surface at a load of 200 g and an indentation duration of 15 s using a Vickers microhardness tester (MICROMET^®^, ADOLPH I. BUEHLER INC, Lake Bluff, IL, USA). An average of at least five readings for each sample was reported in this study. The microhardness results are reported as the microhardness versus the impact angle.

### 2.3. Erodent

**Figure 3:**
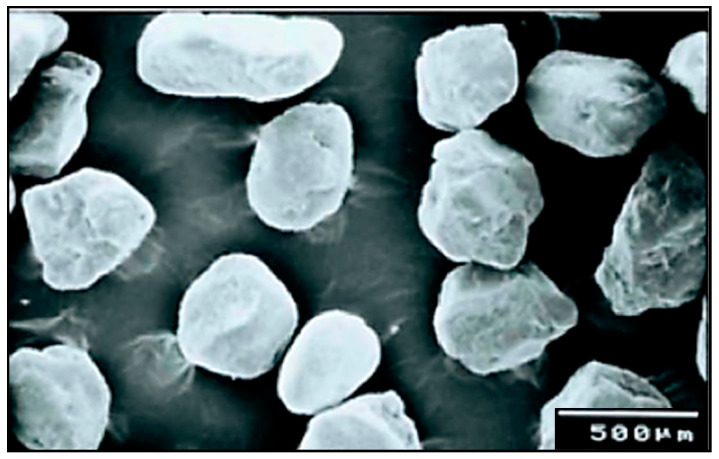
Scanning electron micrograph of SiO_2_ silica sand erodent [51].

Natural SiO_2_ sand with a nominal size ranging from 355 to 500 μm was employed as an erodent to form a solid–liquid slurry with tap water and artificial seawater (tap water + 3.5% NaCl). A scanning electron micrograph of the erodent is shown in Figure 3, and the statistical values of the used SiO_2_ particles are listed in Table 3. The SEM image in Figure 3 shows that the particles’ shapes were relatively block-like and regular.

### 2.4. Slurry Erosion and Erosion–Corrosion Tests Procedure

**Figure 4:**
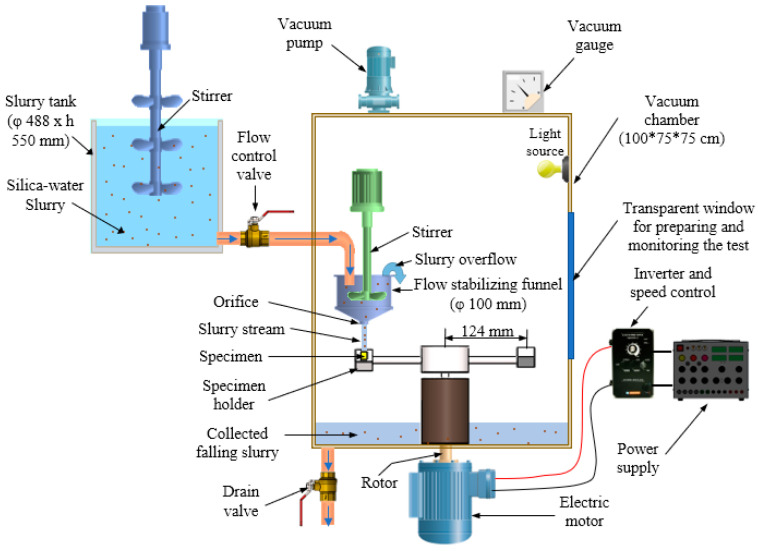
Schematic view of the slurry whirling-arm rig.

**Figure 5:**
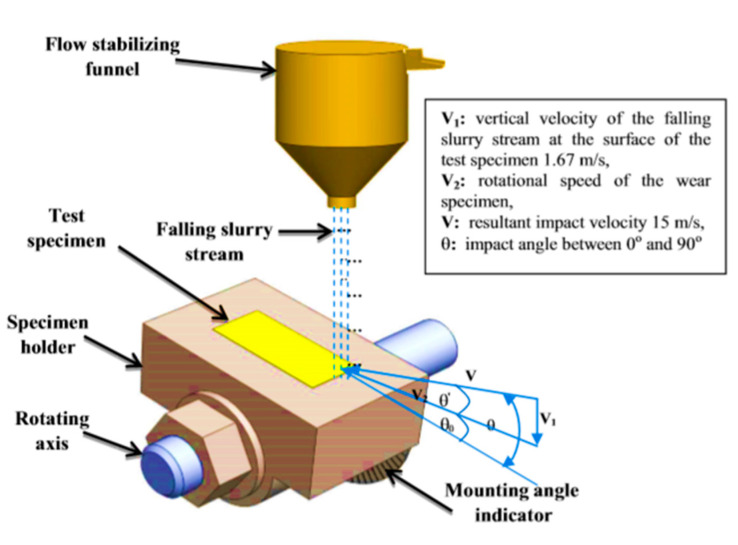
Schematic diagram of the impact angle and impact velocity.

Erosion and erosion–corrosion tests were performed using a whirling arm slurry erosion test rig (WASET) [52], as shown in Figure 4 and Figure 5. The rig was composed of 3 main units: a slurry mixing unit, a slurry test chamber, and a vacuum unit. The details of the used experimental test rig design and performance are provided in [53,54]. In the first set of experiments (erosion tests), the procedure started with a slurry mixing unit consisting of a 25 L cylindrical tank containing 1% sand particles that were added to tap water and mixed via a stirrer, before being passed through a pipe to the slurry test chamber. In the slurry test chamber, the slurry mixture flowed to a funnel with a 3 mm diameter orifice equipped with a stirrer to keep the slurry under suspension. The funnel provided a falling homogenous stable slurry stream at the center of the sample surface. Test specimens were placed on two holders mounted at the ends of two horizontal arms fitted at 180° apart from each other to balance the dynamic forces. The diametric holder-to-holder distance was 248 mm, and samples were positioned 40 mm from the tip of the orifice. An impact angle from 0° to 90° could be adjusted by rotating the sample holder around the arm axis, as shown in Figure 5. The two arms were attached to a brass sleeve firmly tightened to the top end of a vertical whirling shaft which provided balance under high-speed operation and was driven by a variable speed motor. Two samples were tested at the same time, and a single surface with dimensions of 23 mm × 10 mm (see Figure 1) for each sample was exposed to the slurry stream at impact angles of 30°, 45°, 60°, and 90°. To eliminate aerodynamic effects on the slurry stream, the slurry test chamber was evacuated by a vacuum system (up to 28 cm Hg). The second set of experiments (erosion–corrosion tests) followed the same procedure, but samples were exposed to a slurry with seawater (tap water + 3.5% NaCl) instead of tap water. To replenish the consumed slurry during any set of experiments, predetermined amounts of pure water (or seawater) and SiO_2_ sand flowed and were stirred continuously in the slurry tank.

At each test condition, five measurement intervals were carried out, and the weight of the specimen was very carefully measured before and after each interval using a precision balance with an accuracy of ±0.1 mg. Two specimens were tested in each test condition, and the average mass loss value was reported. All of the slurry erosion tests were carried out at an ambient temperature of about 25 °C and a relative humidity of 30–40%.

At the same test time, specimens at different impact angles were not subjected to the same mass of erodent. Therefore, in this test rig, a comparison between the different impact angles was carried out depending upon the mass of the erodent, i.e., all samples at each impact angle should be impacted by the same amount of erodent and not the same test-time. Table 4 illustrates the mass of the erodent that impacts the surface of the sample and the corresponding test time at each impact angle. If the interval mass of the erodent was 28.2 g of SiO_2_ particles, then the test time was 15, 17.5, 21.6, and 30.9 min at impact angles 90°, 60°, 45°, and 30°, respectively. The mass of the erodent and the corresponding test time at each impact angle was determined according to Equation (1) [52]:

(1) $$m_{p}=\left[l\sin(\theta_{o})\;A_{n}+\frac{lCos(\theta_{o})\;Q}{\pi DN}\right]C_{w}\rho_{w}$$

where *θ_o_* is the angle between the surface plane of the specimen and the horizontal plane; *l* is the length of wear specimen surface in *m*; *A_n_* is the area of the orifice in m^2^; *C_w_* is the weight fraction of solid particles in the water; *ρ_w_* is the water density in kg/m^3^; *D* is the rotational diameter of the wear specimen in m; *Q* is the volume flow rate of slurry in m^3^/min; *N* is the rotational speed of the wear specimen in rpm.

## 3. Results and Discussion

### 3.1. As-Built Microstructure

**Figure 6:**
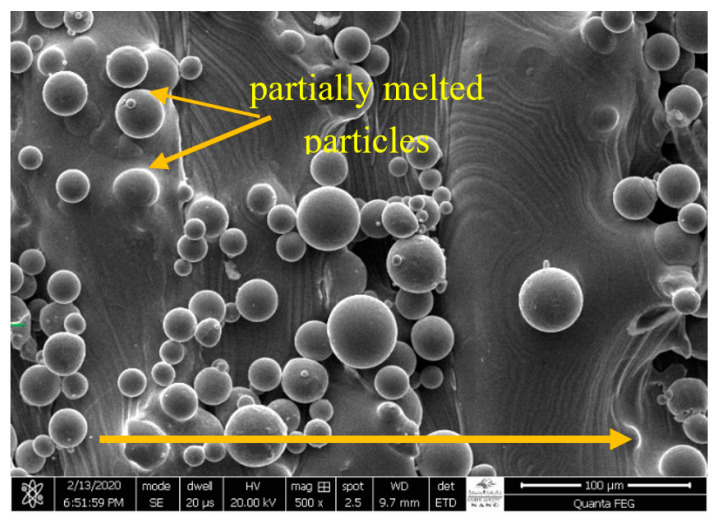
SEM image of the external surface morphology of as-built SLMed Ti6Al4V specimens (arrow shows the building direction).

Figure 6 shows an SEM image of the morphology of the external surface of as-built SLMed Ti-6Al-4V specimens. The image reveals the presence of un-melted and partially melted powder particles adhering to the surface due to the high scan speed and rapid cooling, causing increased surface roughness which is a common characteristic in the SLM process.

**Figure 7:**
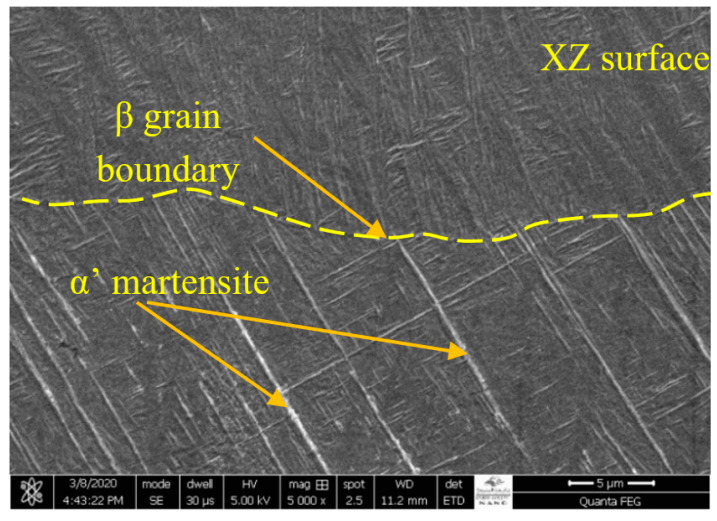
SEM microstructure of as-built SLMed Ti6Al4V.

The microstructure of as-built SLMed Ti-6Al-4V is shown in Figure 7. It can be seen in Figure 7 that the microstructure consisting of a fine acicular α’ martensite originated from the prior β grain boundaries. This microstructure evolved due to the martensitic transformation of the bcc β-phase as a consequence of the inherent rapid heating and cooling of the SLM process. These findings are in accordance with reported work [38,39,40,41,42,43]. As a consequence, the as-built Ti-6Al-4V possesses high hardness and strength, giving it greater wear resistance compared to its as-cast counterpart [55]. SLM process parameters, including the laser type and energy, scan parameters and strategy, powder characteristics, and layer thickness have strong influences on the surface quality and microstructure [41].

### 3.2. Eroded Surface Characteristics and Erosion Mechanisms

**Figure 8:**
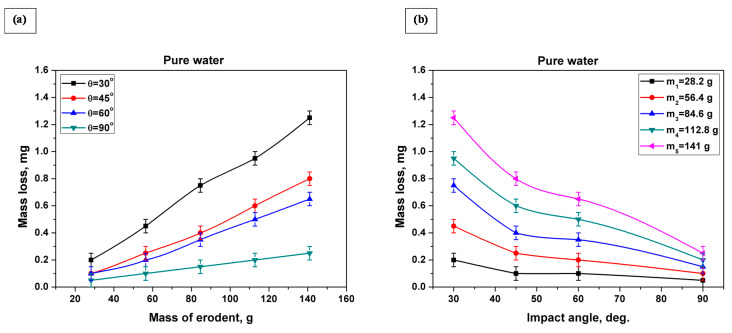
Mass loss of the SLMed Ti-6Al-4V alloy due to slurry erosion in pure water at impact angles 30°, 45°, 60°, and 90°: (**a**) mass loss versus mass of erodent; (**b**) mass loss versus impact angle.

**Figure 9:**
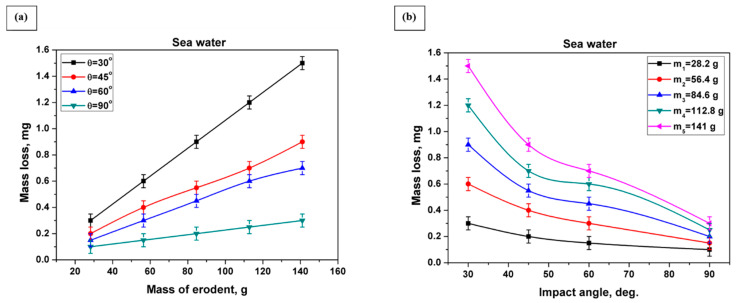
Mass loss of the SLMed Ti-6Al-4V alloy due to slurry erosion in seawater at impact angles 30°, 45°, 60°, and 90°: (**a**) mass loss versus mass of erodent; (**b**) mass loss versus impact angle.

Figure 8a,b and Figure 9a,b show the accumulative mass loss of specimens as a result of slurry erosion tests using pure water and artificial seawater, respectively, at impact angles of 30°, 45°, 60°, and 90°. By increasing the striking mass of the erodent (increasing the test time) on the samples, the mass loss increased dramatically with an almost constant rate for all impact angles—30°, 45°, 60°, and 90°—and for both mediums (pure water and seawater). Also, from Figure 8b and Figure 9b, it is clear that the amount of mass loss decreased as the impact angle increased. The highest mass loss value due to slurry erosion of both mediums (pure water, and seawater) was recorded at an impact angle of 30°, while the lowest value was recorded at an impact angle of 90°.

**Figure 10:**
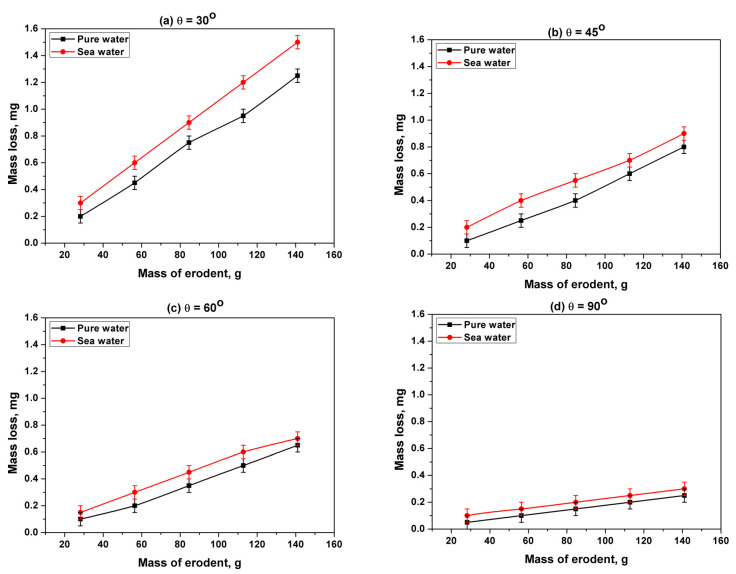
Mass loss of the SLMed Ti-6Al-4V alloy due to slurry erosion in pure water and seawater at impact angles of (**a**) 30°, (**b**) 45°, (**c**) 60°, and (**d**) 90°.

Although titanium alloys have good resistance to corrosion, seawater is considered a severe medium for most commonly used materials, and it influences the amount of material lost due to corrosion. As the corrosion of specimens in pure water is negligible [56], the mass loss due to slurry erosion using pure water is referred to as erosion mass loss (W_E_). The mass loss due to slurry erosion using seawater is referred to as the erosion–corrosion mass loss (W_EC_). The erosion–corrosion mass loss (W_EC_) was found to vary similarly to the erosion mass loss (W_E_) at impact angles of 30°, 45°, 60°, and 90°. At all studied impact angles—30°, 45°, 60°, and 90°—the mass loss due to slurry erosion in seawater was always higher than that due to slurry erosion in pure water, i.e., W_EC_ was higher than W_E_ at all impact angles (see Figure 10). Also, it is clear from Figure 10 that when the impact angle increased, the W_EC_ and W_E_ curves and their error bars approached each other. This indicates that the effect of corrosion decreases as the impact angle increases.

**Figure 11:**
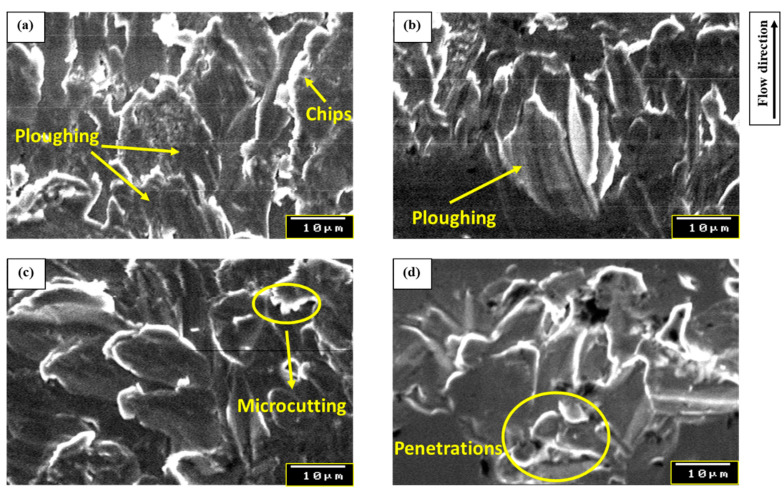
SEM morphologies of the SLMed Ti-6Al-4V alloy due to slurry erosion in pure water and seawater at impact angles of (**a**) 30°, (**b**) 45°, (**c**) 60°, and (**d**) 90°.

The previous results can be explained by considering the slurry erosion mechanism at each impact angle. At relatively small impact angles (e.g., *θ* = 30°), the dominant slurry erosion mechanism is plowing, whereas the impacted solid particle forms a relatively long crater on the surface of the sample with some depth, causing chips to be accumulated on both sides of this crater and in front of it, as shown in Figure 11. When the impact angle increased, the length of the slurry erosion craters decreased. The impacting force has two components, the horizontal impacting force component and the vertical impacting force component, and both of these components work simultaneously to form the slurry erosion crater. At low impact angles, the high horizontal force component causes long craters, while at high impact angles (e.g., *θ* = 90°) the vertical force component causes penetrations, micro-forging, and extrusions on the surface of the specimens [54,57]. In between, at medium impact angles (e.g., *θ* = 45°, and *θ* = 60°) the predominant mechanisms are a mixture of plowing and micro-cutting mechanisms (see Figure 11). Furthermore, because of the high strength and ductility of the SLMed Ti-6Al-4V alloy compared to other materials, such as low alloy steel, the SLMed Ti-6Al-4V alloy has a high resistance for penetration but a weakness for scraping. Therefore, the effect of the vertical impacting force component is insignificant compared with the effect of the horizontal impacting force component. Accordingly, at an impact angle of 30° the mass loss was found to be the highest, while at an impact angle of 90°, the mass loss was the lowest. This suggests that the erosion mechanism is similar to slurry erosion using pure water slurry or sodium chloride water (seawater) slurry [58].

**Figure 12:**
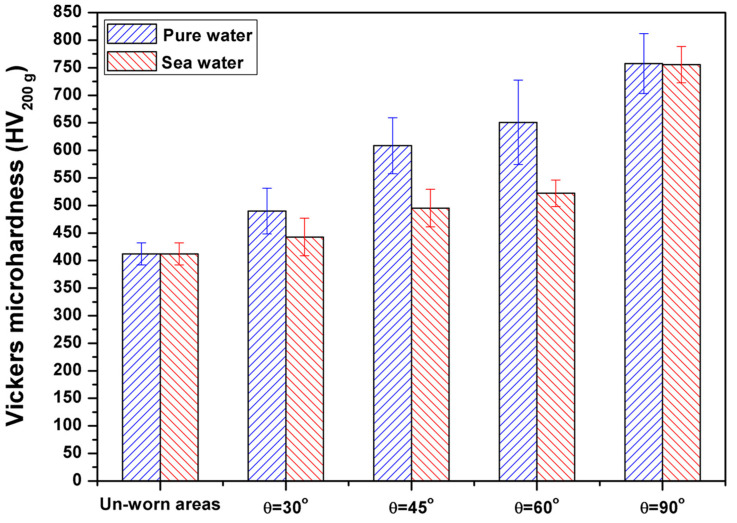
Microhardness values of worn and unworn SLMed Ti-6Al-4V alloy. The worn areas are the result of slurry erosion in pure water and seawater at impact angles of 30°, 45°, 60°, and 90°.

Additionally, through measuring the microhardness along with the slurry eroded pathway in the middle of the surface, it was found that the microhardness value increased when the impact angle increased (Figure 12), unlike the unworn areas that were not exposed to any strikes, which showed the lowest microhardness value (412 HV). The measured microhardness values at impact angles of 30°, 45°, 60°, and 90° were 490, 608.5, 650.8, and 757.6 HV, respectively, for pure water slurry and 442.8, 495.3, 522.3, and 755.7 HV, respectively, for seawater slurry. It is clear from the microhardness results at all impact angles that the increased surface hardness resulted in lower mass losses for both erosive and corrosive tests. A higher microhardness can effectively block plowing, micro-cutting, penetration, and the separation of formed chips. 

Despite the excellent corrosion resistance of the Ti-6Al-4V alloy, its slurry erosion–corrosion resistance in a corrosive environment such as seawater is weaker than that in a pure water environment at all impact angles. The superior corrosion resistance of Ti-6Al-4V alloy is due to the passive oxide film that protects it from corrosive agents such as seawater. However, under the consecutive impacts of sand particles on the surface of the alloy, the formed passive film is destroyed and removed, causing subsequent exposure of fresh active material to seawater.

Natively formed TiO_2_ films on titanium alloys, in general, have poor mechanical properties and they can be easily fractured under slurry erosion conditions or any other mechanical actions. Due to the successive slurry impacts, the distortion of the oxide layer and the reformation of the passive oxide layer processes, which cause a sustained dissolution of the underlying metal, result in gradual consumption of the material [56,59,60,61,62]. Furthermore, the native passive film resistance can change due to the film’s structural changes or changes in the electrical conductivity or ionic components of the film. The formed vanadium oxide V_2_O_5_ on the surface of the Ti-6Al-4V alloy strongly dissolves due to the presence of Cl^−^ ions in the seawater [62,63]. Dissolution of vanadium oxide V_2_O_5_ results in the creation and diffusion of vacancies in the oxide layer of the SLMed Ti-6Al-4V alloy. Therefore, the change in the passive behavior of SLMed Ti-6Al-4V alloy with slurry erosion testing using seawater (contain Cl^−^ ions) may be the cause of the lower microhardness values and high mass losses compared to those of the same material tested in pure water. 

**Figure 13:**
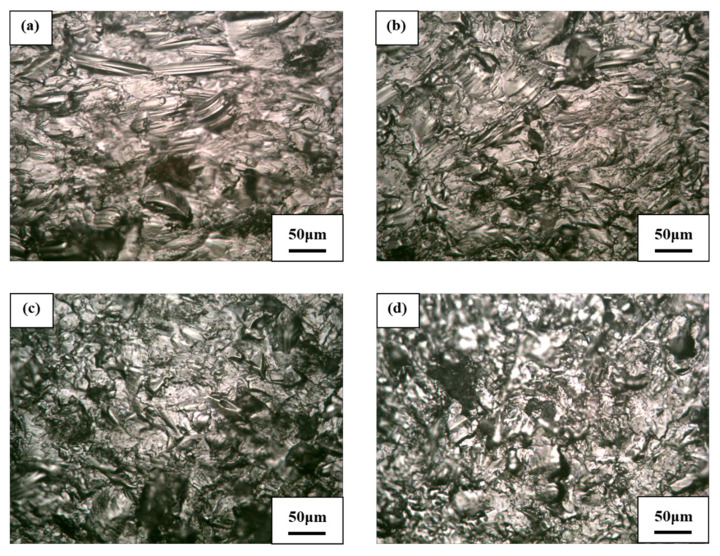
Optical microscopy morphologies of the SLMed Ti-6Al-4V alloy due to slurry erosion in pure water at impact angles of (**a**) 30°, (**b**) 45°, (**c**) 60°, and (**d**) 90°.

**Figure 14:**
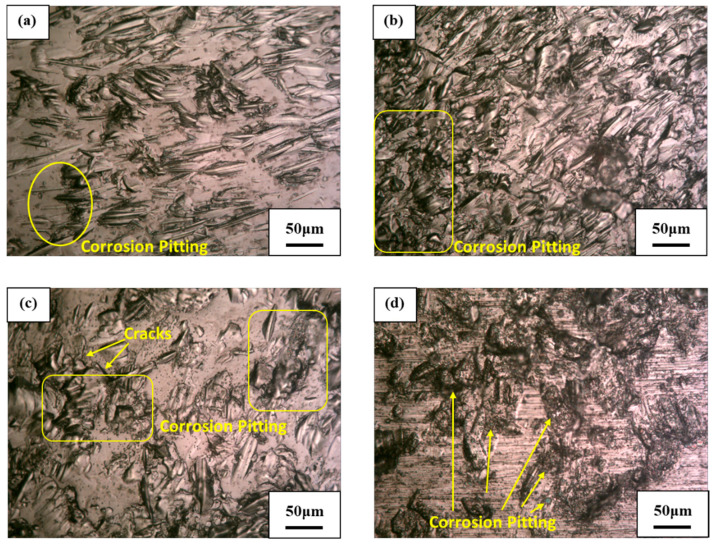
Optical microscopy morphologies of the SLMed Ti-6Al-4V alloy due to slurry erosion in seawater at impact angles of (**a**) 30°, (**b**) 45°, (**c**) 60°, and (**d**) 90°.

The slurry erosion test at a normal impact angle is similar to the wet shot peening process. For both of these tests, the specimens are impacted in the vertical direction with high-speed solid particles. Shot peening increases the strength and durability of the surface and makes it more resistant to many wear types, which may be the cause of the low mass loss of the SLMed Ti-6Al-4V alloy at a normal impact angle (i.e., *θ* = 90°); however, reducing the impact angle from the normal angle causes the strength of the sample surfaces to decrease, which leads to greater mass loss. This further illustrates the behavior of slurry erosion at different impact angles of the SLMed Ti-6Al-4V alloy. Figure 13 and Figure 14 show the optical microscopy images of the solid particles impacting the specimen surfaces at impact angles of 30°, 45°, 60°, and 90° using pure water slurry and seawater slurry, respectively. Figure 13 also shows the large size of micro-distortion that occurred on the surface of the samples as a result of the successive solid particle strikes. These micro-distortions increased slurry erosion resistance and surface roughness. It can also be seen from Figure 14 that small pits over the SLMed Ti-6Al-4V alloy surfaces were observed at all impact angles; therefore, the mode of degradation under the seawater environment is pitting corrosion.

## 4. Conclusions

In this study, the erosion and erosion–corrosion behaviors of as-built SLMed Ti-6Al-4V alloy were examined using pure water slurry and seawater slurry, respectively, at different impact angles. The microstructure of as-built SLMed Ti-6Al-4V appeared as a needle-like martensitic α’ phase. The impact angle was found to dramatically affect the erosion and erosion–corrosion behaviors of SLMed Ti-6Al-4V alloy. The SLMed Ti6Al4V alloy showed ductile erosion behavior, and the maximal mass losses for erosion and erosion–corrosion occurred at an impact angle of 30°. The minimal mass losses for erosion and erosion–corrosion occurred at an impact angle of 90°. SEM investigations of the eroded surfaces of the SLMed Ti6Al4V alloy specimens revealed the dominant erosion and erosion–corrosion mechanisms, such as ploughing, microcutting, and penetrations, at each impact angle. The microhardness increased as the impact angle increased in both erosion and erosion–corrosion tests. The vanadium oxide V_2_O_5_ formed on the surface of the Ti-6Al-4V alloy strongly dissolved due to the presence of Cl^-^ ions in the seawater. Therefore, the mass losses of slurry erosion–corrosion was higher than that of slurry erosion at all impact angles. 

## Figures and Tables

**Table 1:** Chemical composition (wt.%) of used Ti-6Al-4V.

Element	Ti	Al	V	C	Fe	O	N	H	Yttrium
wt.%	Balance	5.50–6.50	3.50–4.50	≤0.08	≤0.25	≤0.13	≤0.05	≤0.012	≤0.005

**Table 2:** Process parameters of selective laser melting (SLM).

Laser Power (W)	laser Spot Size (μm)	Scanning Velocity (m/s)	Layer Thickness (μm)	Hatch Spacing (mm)	Hatching Strategy	Atmosphere
200	75	0.857	60	0.095	Meander	Inert atmosphere (Purged with Argon gas)

**Table 3:** Statistical values are determined from image analysis processing of particle parameters.

Particle Size Range (µm)	Statistical Parameters	Area, A (µm)^2^	Average Dia. (µm)	Width, W, (µm)	Length, L, (µm)	Aspect Ratio = (W/L)	Perimeter, P, (µm)	P^2^/(4πA)
355–500	Mean Median Std. Dev	130,512	395.66	358.12	496.53	0.734	1435.71	1.29
		128,716.2	396.74	356.10	481.85	0.727	1402.37	1.23
		33,737.24	50.35	54.62	84.61	0.126	192.53	0.213

**Table 4:** Test-time corresponding to the impact angle for the same mass of erodent (*m_p_* = 1.8729 g).

Impact Angle *θ*, Deg.	Mass of Erodent *m_p_*, (g)	Corresponding Test Time t, (min.)
15	1.8729	4.12
30	1.8729	2.06
45	1.8729	1.44
60	1.8729	1.17
75	1.8729	1.04
90	1.8729	1.00
